# Supplementary material for: Are carbon emissions trading and green financial instruments synergistic? -Comprehensive quantitative research based on content analysis
Source: PLoS One. 2024 Mar 7;19(3):e0298601. doi: 10.1371/journal.pone.0298601 (PMC10919601; doi:10.1371/journal.pone.0298601)
Supplement: S1 Data — (ZIP) [file pone.0298601.s001.zip › Data/地级市Z_F工作报告.zip!/╔╜╢1⁄2/╤╠╠¿╩╨/└┤╫╘╣1⁄2╓┌║┼í╛┬φ┐╦╩2╛▌═°í┐_2010.docx]

各位代表：

现在，我代表市人民政府向大会报告工作，请予审议，并请市政协委员和其他列席会议的同志提出意见。

一、2009年政府工作回顾

2009年是新世纪以来我市经济社会发展最为困难的一年。一年来，面对历史罕见的国际金融危机，全市上下在中共烟台市委的正确领导下，坚定信心、团结奋斗，迎难而上、共克时艰，扎实推进保增长、保民生、保稳定各项工作，全市改革开放和现代化建设在非常时期取得了新的重大成就。

——积极应对金融危机冲击，经济保持平稳较快增长。认真贯彻党中央、国务院和省委、省政府应对危机的决策部署，实施了一系列积极有效的政策措施，较好地完成了保增长任务目标。初步核算，全市实现生产总值3728.7亿元，比上年增长13.5%；地方财政收入完成189.1亿元，增长13.8%。各级各部门抢抓扩大内需的政策机遇，积极争取资金、项目和政策，深入开展“项目推进年”和“集中开工月”活动，增强了投资对发展的拉动作用。全市固定资产投资完成2222亿元，增长25.1%。由国家批准的重大项目投资达到1000亿元以上。潮水国际机场正式奠基，并被列为全国机场改革试点单位；蓝烟铁路电气化改造主体工程完工，龙烟铁路开工建设，青烟威荣城际铁路开工准备就绪；莱山至海阳高速公路前期工作进展顺利，三条疏港高速、海阳跨海大桥等工程建设加快推进；西港区防波堤一期和20万吨级航道工程竣工。长期制约我市发展的交通瓶颈将得到历史性突破。全面落实国家鼓励消费的各项政策，积极开展“消费推动年”活动，出台了促进房地产市场平稳健康发展的18项措施，城乡市场保持良好势头。全市社会消费品零售额完成1220亿元，增长19.2%。全年促销家电下乡产品29.3万台，汽车、摩托车下乡7.5万辆。千方百计帮助企业渡过难关，先后两次停止和取消212项行政事业性收费，降低4项社保费率，推行了增值税转型改革，全年共为企业减轻负担21.4亿元。设立了中小企业发展专项扶持资金，成立了3家小额贷款公司，多次组织银企对接洽谈活动，新引进7家金融机构入驻烟台。年末金融机构各项贷款余额比年初增加604亿元，是上年新增额的3.2倍。

——加大结构调整力度，三次产业协调发展。组织引导企业和各类市场主体，充分利用金融危机形成的倒逼机制，转变发展方式，推动产业结构优化升级。三次产业结构由上年的8：60.9：31.1调整为7.7：60.5：31.8。围绕做大做强先进制造业，出台了八个行业调整振兴规划，实施了一批规模大、层次高、产业关联度强的重大项目。海阳核电全面开工，烟台化学工业园启动建设。全市规模以上工业增加值、利税、利润分别增长16.1%、15.4%和16.5%。高新技术产业产值完成3771亿元，占规模以上工业的比重达到42%。全市主营业务收入过10亿元、利税过亿元的企业均超过100户。新增国家级工程技术研究中心和企业技术中心2家、中国驰名商标9件，荣获首届山东省省长质量奖1项。继续淘汰落后产能，全年关闭13条立窑水泥生产线，二氧化硫和化学需氧量排放分别下降2%和6%，成为全省唯一连续三年完成节能降耗目标的市。围绕加快发展服务业，制定了《全市服务业发展意见》，加大服务业重点城区、重点园区、重点项目、重点企业和特色街区“五大载体”培育力度。全市服务业完成增加值1187.6亿元，增长15.3%。服务业提供的地方税收达到71.5亿元，占全部地方税收的47%。旅游经济逆势上扬，接待海内外游客2803万人次，完成旅游总收入273.7亿元，分别增长17.7%和19.8%。大南山生态景区、雨岱山渔人码头等旅游项目全面推进，旅游大世界、皇冠假日酒店等旅游服务设施投入运营。成功举办了第三届烟台国际葡萄酒节、中国绿色食品博览会、中国教学仪器设备博览会、北方旅游交易会等各类展会80个，参会人数达到264万人次。烟台直飞台湾航班获得批准，新开通烟台至美国、荷兰的全货机航班。全市港口货物吞吐量完成1.69亿吨，增长15.1%。烟台机场旅客吞吐量完成209.5万人次，货邮吞吐量完成3万吨，分别增长22.7%和15.8%。围绕巩固提升现代农业，全面落实强农惠农政策，发放各类涉农补贴3.3亿元。粮食生产连续七年获得丰收，小麦单产创历史最高纪录。肉蛋奶产量达到96万吨，水产品产量达到184万吨，水果产量达到439万吨。我市获得“中国食品名城”和“中国绿色食品城”称号，新增6个国家地理标志证明商标和4个国家地理标志保护产品。

——千方百计稳定外需，改革开放纵深推进。出台了鼓励外经贸发展的11项措施，组织企业参加广交会、津洽会等大型经贸活动，帮助企业巩固客户、拓展市场，有力抑制了出口下滑的被动局面。全年完成外贸进出口总额342.9亿美元，下降2.1%。其中出口198.3亿美元，下降3.9%，降幅低于全国、全省10个百分点以上，在首批14个沿海开放城市中降幅最低。加大招商力度，创新招商方式，利用外资的层次和水平有了新的提高。全市实际使用外资10.9亿美元，增长2.6%；注册外资17.2亿美元，增长2.8%。新引进8个世界500强项目，8户企业实现外资并购，132户外资企业实施了增资扩股。面向国内外500强、中央直属企业、大专院校和科研院所，积极开展“三个引进”工作，共签约43个项目，协议总投资860亿元。 “走出去”战略步伐加快，对外经济技术交流与合作健康发展。外事侨务工作取得新进展，与韩国安山市缔结了友好合作城市关系。经济技术开发区的开放龙头作用持续增强；保税港区获得批准，成为全国第一家由出口加工区和港口整合形成的开放园区；出口加工区B区规划顺利调整；高新区新一轮规划建设全面启动。各领域改革进一步深化。政府机构改革稳步推进，医药卫生体制改革开始启动。省管县财政改革试点、集体林权制度改革取得积极进展。农村土地承包经营权流转总面积38.9万亩，流转率达到6.7%。价格改革不断深化，市区规划执法、食品药品监管和文化市场管理体制进一步理顺。烟台港集团等12户市属国有企业完成改革改制任务，烟台杰瑞等4家公司上市申请获得通过。民营经济健康发展，增加值达到3011亿元，增长14.5%。新登记个体工商户2.94万户，新注册私营企业7520家。

——城乡建设力度加大，基础设施日益完善。强化规划在城市建设中的龙头引领作用，城市规划区控规覆盖率达到83.6%。中心市区实施了174项城建重点工程，年度投资达到120亿元。备受全市人民关注的烟台新火车站、文化中心投入使用，我市又添新的标志性建筑。北马路和毓璜顶医院周边改造、红旗中路“一隧两桥”建设等工程加快推进，养马岛至三山岛全长200公里的滨海大道全线贯通，山海南路、芝罘岛西路等16条市区道路及同三高速福山立交、莱山立交等一批路桥工程建成通车。迎春大街商业区开工建设，银座商城、天和大厦主体完工。开展了为期150天的市区环境综合整治活动，城市形象有了新改观。城市功能进一步完善，全市新增集中供热面积621.7万平方米，新增天然气用户5.7万户。新建改建4处污水处理厂和4处垃圾处理厂，中心市区雨污分流工程按计划完成。将牟平公交纳入市区公交体系，公共交通网络更加健全。新农村建设步伐加快，启动了为期三年的农村住房建设和危房改造工程，新建改造农村住房457万平方米。新增城乡生活垃圾一体化处理村(居)615个，新发展沼气用户3万户。改造硬化农村公路515公里,新建改造740个村的自来水工程。完成了3座中型水库主体工程和209座小型水库除险加固任务。加大土地开发整理力度，继续保持耕地总量动态平衡。新增造林绿化面积21.7万亩，全市森林覆盖率达到39%。

——民生状况持续改善，社会事业全面进步。城市居民人均可支配收入达到21125元，农民人均纯收入达到8642元，分别增长9.2%和8.9%。新增城镇就业14.8万人，其中失业职工再就业3.9万人，新增农村劳动力转移就业3.5万人，城镇登记失业率控制在3.3%。为民服务十件实事全部完成。社会保障水平进一步提高，福山、招远、开发区被列入全国首批新型农保试点，全市农民养老金提高到年人均600元，企业退休人员养老金月人均达到1340元，城乡低保标准分别提高到月人均333元和年人均1544元。全市新提供经济适用房4830套，新增加廉租住房保障户955户。城镇居民基本医疗保险覆盖率达到85%，未成年参保居民政府补助标准提高到每人每年80元。实施农村中小学校舍改造、教学仪器更新和“两热一暖一改”工程，将农村小学生和初中生人均公用经费分别提高到400元和600元。继续开展“爱心捐助”活动，全年发放救助金7906万元，救助困难群众24.5万人次。科技事业成果丰硕，中科院烟台海岸带研究所正式挂牌，清华(烟台)科技园开工建设。18个项目列入国家863计划、科技支撑计划和重大专项，5项成果获得国家科技奖，申请国家专利6467件。深入实施人才强市战略，新引进海内外各类人才2.3万人。文化市场日趋繁荣，国庆60周年系列活动丰富多彩。成功组织了第十一届全运会烟台赛区各项赛事，烟台健儿在全运会上取得13.5枚金牌的优异成绩，我市获得“动力之都”称号。城市社区卫生服务机构覆盖率达到95.8%，完成20处乡镇卫生院和1120所村卫生室改造，新农合全面覆盖农村居民，甲型H1N1流感、手足口病等重点传染性疾病得到有效防控。气象灾害预警机制更加健全，成功抵御了特大暴雨、暴雪灾害。人口和计划生育工作保持先进水平，老龄、妇女、儿童、红十字和残疾人事业得到巩固加强，广播电视、新闻出版、防震工作、史志档案、双拥共建、人民防空、民族宗教、国防后备力量建设等各项社会事业取得新的进步。支援四川地震灾区恢复重建工作继续在全省保持领先位置，帮扶栖霞、支持长岛工作扎实推进，援藏、援疆和帮扶定陶工作取得新的成效。

——民主法制建设继续加强，和谐稳定形势更加巩固。进一步完善政府决策机制，加强公务员队伍建设，干部队伍素质和依法行政水平有了新的提高。认真办理人大代表建议和政协提案，与民主党派、工商联、无党派人士和人民团体的联系更加紧密。发展环境日趋优化，荣获“共和国60年最具投资潜力城市”称号。反腐败斗争深入推进，廉政建设成效明显。安全生产形势稳定，应急管理水平不断提高。我市再次荣获全国文明城市称号，获得全国社会治安综合治理优秀城市“五连冠”。

各位代表！刚刚过去的2009年，是我市发展史上极为特殊、极不平凡的一年。这一年，是我市不畏艰难险阻，奋力应对复杂局面、经受各种严峻考验的一年。在百年不遇的国际金融危机面前，全市上下牢牢把握“昂扬精神迎挑战，科学务实谋发展”的工作主基调，坚持把保增长作为经济工作的首要任务，迎难而上、沉着应对、积极作为，变压力为动力，化挑战为机遇，在短时间内有效遏制了经济增速下滑趋势，率先实现了经济形势总体回升向好。在抵御金融危机冲击的斗争中，我们既取得了经济逆势回转的工作业绩，又积累了在复杂环境中推动又好又快发展的智慧和经验。这一年，是我们采取非常措施，营造内生发展动力、推动经济稳步前行的一年。全面落实扩内需、保增长政策，出台了40多项帮扶企业、促进发展的政策措施，争取到中央、省扩大内需资金30.7亿元，实施了一批事关全局和长远发展的重大项目，办成了一批多年想办而没有办成的事情。各级各部门全力优化发展环境，设身处地为企业排忧解难。一系列行之有效的政策措施，极大提振了全市上下的发展信心，突破了影响发展的种种制约，推动我市经济在前进中崛起，使应对金融危机冲击的过程成为推动经济发展上水平的过程，成为为长远发展打基础的过程。这一年，是各级创新工作思路，科学谋划未来发展、培育竞争新优势的一年。着力推动产业结构、区域结构的战略性调整，积极实施山东半岛蓝色经济区、胶东半岛高端产业聚集区、黄河三角洲高效生态经济区建设“三大战略”，快速启动新一轮城镇化发展战略，加快培育和发挥龙头板块、支柱产业、骨干企业的引领带动作用，全市经济在发展中调结构，在调整中转方式，迈出了向集约化、高端化、特色化转型升级的历史性步伐。这一年，是全市上下团结奋斗，民心民力高度凝聚、发展热情更加高涨的一年。在严峻的困难和挑战面前，各级各部门始终保持清醒头脑，坚定必胜信念，以时不我待的精神和坚韧不拔的意志，瞄准目标、敢打敢拼、苦干实干，形成了聚精会神搞建设、一心一意谋发展的强大合力。各企业在异常艰难的情况下，积极顺应形势变化，千方百计调结构、保市场、扩外需，坚持不裁员或少裁员、不减薪或少减薪，既推动了经济发展，又维护了社会稳定，展现了高度的社会责任感和新时期的企业家风采。全市人民在困难中更加紧密地团结在一起，同舟共济、众志成城，构筑起应对冲击、抵御风险的坚强防线，凝聚成共克时艰、励精图治的伟大力量。这是我们战胜困难、夺取胜利的宝贵财富，必将有力推动我市改革开放和现代化建设事业取得更加辉煌的成就！

各位代表！过去一年我市经济社会发展所取得的成绩极其不易。这是上级党委、政府和中共烟台市委正确领导、科学应对的结果，是市人大、市政协有效监督、支持参与的结果，凝聚了全市人民的智慧、心血和汗水。在此，我代表市人民政府，向全市人民，向人大代表、政协委员，向各民主党派、工商联、各人民团体和各界人士，向中央、省属驻烟单位，向驻烟解放军、武警官兵，致以崇高的敬意！向所有关心支持烟台发展的香港和澳门特别行政区同胞、台湾同胞、海外侨胞、国际友人，表示衷心的感谢！

在总结成绩的同时，我们也清醒地看到，我市经济社会发展还存在一些亟待解决的困难和问题，主要表现为：经济平稳增长的基础还不牢固，发展方式较为粗放，服务业发展滞后，经济结构调整任务艰巨；民间投资跟进不快，消费潜力还没有充分激活，扩大开放的难度仍然很大；自主创新能力不足，部分企业特别是中小企业生产经营仍很困难；财政增支减收因素较多，收支平衡压力加大；农民持续增收的难度较大，部分城乡居民的生活还很困难；就业形势依然严峻，社会保障、教育医疗、社会治安、安全生产、环境保护等方面还存在一些问题；部分干部作风不实、工作效率不高，腐败现象还时有发生，政府公共服务水平有待进一步提高，等等。这些困难和问题已引起我们的高度重视，并采取扎实有效的措施认真加以解决。

二、2010年工作预期目标

今年是实施“十一五”规划的最后一年。做好今年的政府工作，对于夺取应对国际金融危机冲击的全面胜利、为实施“十二五”规划奠定良好基础具有十分重要的意义。

综观国内外经济形势，挑战与机遇并存，机遇大于挑战。当前，国际金融危机影响仍在延续，发达国家消费疲软、美元持续贬值、贸易保护主义不断加剧，世界经济复苏将经历缓慢曲折的过程。我国经济回升的基础还不牢固，结构性矛盾仍很突出，外需不足的问题短时间内难以解决。同时也应看到，国际金融市场渐趋稳定，日韩、港台等我市主要贸易伙伴经济回升的步伐加快。我国已进入工业化、城镇化的中期加速阶段，中央将保持宏观政策的连续性和稳定性，继续实施积极的财政政策和适度宽松的货币政策。我市在“三大战略”中处于重要位置，一批支柱产业加快膨胀，一批骨干企业迅速成长，一批影响深远的重大项目正在快速推进，结构调整与自主创新迈出新的步伐，多年积累的发展能量使我市经济的抗风险能力和可持续发展能力不断增强，具备了良好的发展条件和充足的内在动力。只要我们抢抓机遇、发挥优势，坚定信心、迎难而上，就一定能够战胜困难、经受考验，一定能够开创烟台经济社会发展的新局面。

各位代表！2010年是我市发展全面上台阶、实现新跨越的一年。全市生产总值将突破4000亿元，地方财政收入突破200亿元，城乡居民储蓄突破2000亿元，规模以上工业主营业务收入突破10000亿元。虽然前进的道路上仍然充满风险与挑战，但站在新的历史起点上，完成惠及650万烟台人民的发展大业，我们信心满怀。新的一年，我们要全面贯彻党的十七大和十七届三中、四中全会精神，以邓小平理论和“三个代表”重要思想为指导，深入贯彻落实科学发展观，认真执行国家宏观调控政策，以转变发展方式、调整经济结构为主攻方向，着力提高经济增长的质量和效益，加快推进改革开放和自主创新，积极促进区域协调和城乡统筹，高度重视改善民生和社会稳定，努力实现经济平稳较快发展，全面完成“十一五”规划目标任务。

今年经济社会发展的主要预期目标是：全市生产总值增长11%；地方财政收入增长12%；固定资产投资增长20%；社会消费品零售额增长16%；实际使用外资与去年持平，外贸进出口增长5%；城市居民人均可支配收入和农民人均纯收入均增长8%；城镇登记失业率控制在3.5%以内；人口自然增长率控制在2.15%。以内；万元生产总值能耗、二氧化硫和化学需氧量排放完成“十一五”规划目标。

三、推动科学发展

坚持把转方式、调结构作为深入贯彻落实科学发展观的重要目标和战略举措，着眼于增强发展的稳定性、协调性、平衡性和可持续性，以实施“三大战略”为主抓手，统筹推进经济发展、结构调整、对外开放和城乡建设，打造蓝色经济先行区，建设高端产业示范区，构建高效生态产业体系。

(一)全面贯彻宏观调控政策，进一步巩固经济回升势头。

抓好重点项目建设。继续实施“项目推进年”活动，推进总投资5305亿元的189个市级重点项目，年度完成投资938亿元。加快建设海阳核电、潮水国际机场、龙烟铁路、青烟威荣城际铁路、烟台港西港区、莱山至海阳高速公路、三条疏港高速、华电国际莱州电厂等30个重大能源交通项目，做好石油储备基地及原油码头、城市轻轨、龙青高速龙口至莱阳段、东南部热电等项目前期工作；集中培育宝钢精密钢管、高端铝合金产业园、长链二元酸等65个重点制造业项目；实施好新三站市场、振华国际广场、新天地都市广场等一批服务业和社会文化项目，以重点项目建设带动投资适度较快增长。

努力提高投资质量。调整优化投资结构，加大对重大基础设施、先进制造业、现代服务业、高新技术、节能减排、农业农村、民生和社会事业等领域的投入，坚决防止新上高耗能、高污染和低水平重复建设项目。全面落实国家和省鼓励民间投资政策，放宽市场准入，吸引社会资本参与投资建设。加大项目储备力度，策划论证一批对结构调整和产业升级具有引领作用的大项目、好项目。坚持集约节约用地，盘活闲置和低效土地，扩大城乡建设用地增减挂钩试点范围，保障经济发展和项目建设用地需求。坚守耕地红线，严肃查处各类违法违规用地行为。

扩大城乡居民消费。继续开展“消费推动年”活动，落实国家促进消费各项政策。提高城乡居民收入水平，增强居民特别是低收入群众的消费能力。引导房地产业平稳健康发展，加快中低价位、中小型普通商品房建设，支持居民自住房需求，鼓励发展商业、文化、旅游等产业地产。做好家政服务、早餐示范、放心肉工程、市场监测信息平台建设等便民利民消费工作，实施一批农贸市场改造、社区商业项目。发展夜间经济，培育文化娱乐、体育健身等新的消费形式。鼓励和扶持农村消费，组织实施“万村千乡市场工程”，搞好双百市场、农超对接、下乡产品流通网络、农资流通体系建设。扩大家电下乡和汽车、摩托车下乡规模，落实好以旧换新政策，不断繁荣消费市场。年内，完成社会消费品零售额1415亿元，销售家电下乡产品15万台以上，汽车、摩托车下乡5万辆。

强化财政金融保障。落实积极的财政政策，继续争取中央新增投资项目和国家、省各类扶持资金，加大财政对结构调整、科技进步和民生领域的投入。强化财源建设，培育新的财源增长点，加强税收征管和非税收入管理，促进财政收入平稳较快增长。优化财政支出结构，严格控制一般性开支，努力降低行政成本。深化财政体制改革，稳妥推进省管县财政改革试点工作。继续执行适度宽松的货币政策，引导金融机构把握好信贷投放节奏，增加对重点产业和薄弱环节的信贷投放，加大对农业和中小企业的信贷支持。推进银企、银保合作，搞好村镇银行和小额贷款公司试点。鼓励企业上市融资、集合发债，扶持发展创业投资，加强投融资平台建设。支持金融机构健康发展，争取更多的外资银行、证券公司、信托投资公司等金融机构在我市设立分支机构。

(二)着力推进经济结构调整，进一步提高发展质量效益。

调整振兴工业经济。深入实施汽车等八个行业调整振兴规划，推动信息化与工业化融合，运用先进适用技术改造传统产业，提升自动化、智能化和管理现代化水平，打造一批具有国际竞争力的现代产业基地。搞好通用东岳新增30万辆轿车、丛林轻量化汽车等项目建设，引进和培育汽车零部件企业，发展汽车产业基地。加快富士康工业园、清华(烟台)科技园建设，壮大电子信息产业基地。加紧建设烟台化学工业园，抓好安邦石化一期等工程，建设生态型现代石化产业基地。推进大宇造船、京鲁船业二期和来福士海洋工程等项目，培植船舶配套产业链条，打造临港造船产业基地。着力培育高品质特钢冶金基地、核电设备生产基地，积极申报国家级软件产业基地和生物产业基地。继续实施“3·50工程”，坚持不懈地培强做大龙头骨干企业，加快推进工业企业“退城进园”，实施好90个单体投资过亿元的技改项目，打造千亿元产业集群、百亿元龙头企业和十亿元骨干企业“三个方阵”。年内，规模以上工业增加值达到2600亿元，增长13%。力争主营业务收入过百亿元企业达到10户以上，过10亿元企业达到110户以上。大力实施品牌战略，力争再创一批驰名商标和名牌产品。

培育高新技术产业。按照“高端、高质、高效”的原则，大力发展高端产业和产业链条高端环节，着力构建国际先进、优势集中、富有特色的高新技术产业体系。把握金融危机背景下世界科技发展、产业调整的新趋势，立足我市资源特点和产业基础，科学布局和大力发展新能源、新材料、生物医药、海洋科技、航天航空等战略性新兴产业。以建设创新型城市为目标，把科技创新作为转方式、调结构的中心环节来抓，完善鼓励创新的政策体系，加快创新型企业建设，推动“烟台制造”向“烟台创造”跃升。年内，高新技术产业产值完成4200亿元以上。加强科技创新平台建设，推动产学研结合，推进中科院海岸带研究所、山东综合技术转化烟台中心、烟台中集海洋工程研究院、烟台留学人员创业园等载体建设，争取创建省级以上工程技术研究中心或重点实验室2个、企业技术中心6个。开展技术创新标准化示范市建设，争创一批国家级、省级科技奖励成果。搞好知识产权的创造、保护和运用，争创全国知识产权示范城市。强化人才队伍建设，实施高端人才引进计划，形成以产业聚人才、以人才促发展的良性循环。

突破发展现代服务业。高度重视旅游业的综合拉动作用，努力把旅游业培育成我市新的战略性支柱产业。以建设最佳海滨休闲度假目的地为目标，积极开发海滨度假游、葡萄酒文化游、乡村民俗游、黄金游、温泉游等特色旅游项目，规划建设旅游集散中心、旅游商品研发基地和自驾车旅游营地，搞好城市旅游形象整体宣传和区域协作。加快提升会展业。以建设国际性会展名城为目标，筹办好第四届烟台国际葡萄酒节、第十一届国际果蔬?食品博览会、中国国际美食节等会展活动。繁荣发展商贸餐饮业。抓好商贸骨干企业和大型专业市场建设，力争年内形成2个以上销售额过百亿元、20个过10亿元的企业和市场。大力发展现代物流业。引进国内外知名物流企业，集中培育6个物流园区，推动中韩车载物流项目，构建现代化物流设施、物流信息和物流政策三大平台。年内，全市港口货物吞吐量达到1.8亿吨，集装箱吞吐量突破150万标箱。健全服务业政策保障机制和投入支撑体系，充分发挥服务业引导资金作用，加快服务业“五大载体”建设。引导金融机构开发适应服务企业需求的金融产品，支持社会资金和外资参与服务业发展。发挥芝罘区在服务业发展中的引领作用，鼓励中心市区发展高端服务业，加速生产要素聚集，进一步促进城市繁荣。

提高现代农业发展水平。加强农田水利基本建设和基本农田保护，加大中低产田改造和粮油高产创建力度。实施农业品牌化发展战略，调整优化农业结构，大力发展优质高效农业。搞好农产品科技创新中心试点，促进农业新技术新成果推广应用。培育无公害、绿色、有机和设施农业，完善农产品质量检验检测体系，推进畜牧、水产品规模化、标准化养殖，加强重大动物疫病防控，加快农业机械化步伐。开展百强农业龙头企业创建活动，发展壮大农村专业合作组织，纵深推进农业产业化。积极发展农村二三产业，通过放开户籍限制推动农民向城镇转移，多渠道增加农民收入。深化土地承包经营权改革，健全农村土地流转机制。加快供销合作社改革和发展步伐，构建农村现代经营服务新体系。广泛开展农产品促销活动，畅通农产品销售渠道。

促进民营经济转型升级。实施民营经济“新特优”工程，支持小企业创业发展和中小企业技术创新。推进公共服务平台建设，为中小企业创业创新提供产品研发、检验检测、技术推广等服务。年内，每个县市区都要建立1-2个公共服务平台。引导中小企业与大型企业联动发展，鼓励中小企业通过联合重组做大做强。全面落实扶持企业发展的各项政策，加强信用担保体系建设，帮助企业特别是中小企业克服困难、健康发展。年内，全市民营经济完成增加值和上缴税金均增长12%以上。

大力发展低碳经济。研究出台我市低碳经济发展意见，扶持发展高效低耗产业。强化节能减排目标责任制，加强重点用能企业监管，加快淘汰落后产能，推动节能环保技术与产品应用。年内，关闭14条立窑水泥生产线，推广50万只高效照明产品。依法强化公共机构节能管理，提高能源使用效率。发展循环经济，完善再生资源回收利用体系，提高资源综合利用水平。继续实施蓝天、碧水、青山、绿地“四大生态工程”，建设资源节约型、环境友好型城市。强化重点污染源环境安全监管，狠抓减排重点工程，推进套子湾二期等6个污水处理厂建设，实施雨污分流及管网配套工程，建设改造4个无害化垃圾处理项目。抓好海洋环境保护和生态修复，推进海洋特别保护区建设，开展龙口湾、莱州湾、丁字湾等8个集约用海区域总体规划编制。全面启动集体林权制度改革，促进现代林业健康发展。开展“全国绿化模范城市”创建活动，打好“三年大造林”攻坚战，确保完成造林24万亩，全市森林覆盖率达到40%以上。

(三)深入实施外向带动战略，进一步提升对外开放水平。

推动出口稳步回升。坚持开拓国际市场和扩大国内市场并举，落实国家鼓励外贸发展的各项政策，加快我市由对外贸易大市向强市转变。深入实施市场多元化战略，稳定传统市场，开发新兴市场。抓住中国——东盟自由贸易区全面启动的机遇，扩大对东盟的出口。提高机电产品和高新技术产品出口比重，争创国家级、省级出口创新基地。加快农产品等产品质量安全示范区建设，提高出口产品附加值和竞争力。重点扶持100户骨干出口企业，培育一批拥有核心技术、自主知识产权和自有品牌的出口企业。积极发展一般贸易和服务贸易，促进加工贸易转型升级。加强贸易平台建设，扶持1000户外贸企业开展电子商务。扩大紧缺资源、先进技术和关键设备进口。完善产业损害预警机制和快速反应机制，积极应对国际贸易壁垒。进一步完善大通关体系，为外贸企业发展创造良好的外部环境。

创新招商引资方式。开展“招大引强年”活动，围绕大企业、大项目实施重点招商。加大产业招商力度，精心策划一批重大引资项目，引导外资投向高新技术产业、先进制造业、现代服务业和基础设施领域。提升专业招商的层次和水平，策划实施一系列主题鲜明、形式灵活的小分队招商，发挥好海外招商顾问、国内重点城市商务代表的作用。鼓励外资以并购方式参与市内企业兼并重组，支持外资企业增资扩股，推进基金招商和境外上市，探索跨境换股、交叉持股等引资新途径。拓展引资市场，加强与国际友城的交流，深化对日韩的经济合作，强化对港台的招商工作，突破欧美招商项目。加大“三个引进”力度，积极承接高端产业转移和高新成果产业化项目。推进服务外包基地建设，组织开展“外包企业烟台行”活动，培育一批具备国际资质、拥有自主品牌的服务外包企业。实施“走出去”战略，推动工贸合作区等现有境外投资项目扩规模、上档次，鼓励更多有条件的企业到国外开展资源开发和工贸合作，并购优质资产、品牌和技术，拓展新的发展空间。

促进园区经济升级发展。发挥经济技术开发区在对外开放中的引领作用，以大型骨干企业为龙头，带动电子、汽车、船舶、现代化工等产业集群膨胀发展。全面启动保税港区规划建设，尽快通过国家验收，及早发挥开放前沿高地的政策优势。高标准推进高新区建设，强化基础设施配套，大力发展高端产业，打造一流的创新型科技园区。加快各类省级园区转型升级进程，营造园区发展新优势。

(四)加快推进城镇化步伐，进一步推动城乡一体化发展。

优化城镇发展布局。依托我市城镇和产业布局的基础和特点，从中心市、城市群、发展轴三个层面，构筑市、县、乡、中心村四位一体的新型城镇化发展格局。中心市，就是以芝罘、莱山、福山、牟平和开发区、高新区为主体的中心城市，构建全市经济社会和城镇化发展的核心带动区。城市群，就是以蓬莱、长岛、龙口、招远、莱州为主体的北部沿海城市群和以海阳、莱阳、栖霞为主体的南部交通干线沿线城市群，分别构建新型城镇化发展的综合示范区和优化提升区。发展轴，就是以威乌高速、同三高速和规划建设的莱山至海阳高速为轴线，辐射城乡干线周边镇村，构建新型城镇化发展的潜力增长区。

做大做强中心城市。实施“沿海建城、沿河开发、环湾改造、依山布局”的城市发展战略，高档次推进大南山、夹河、辛安河“一山两河”建设，推动芝罘岛到养马岛沿海一线开发，打造城市新景观，建设特色文化旅游区和国际化商务服务区，带动六区快速融合。加快毓璜顶医院周边、北马路拓宽改造二期和迎春大街、幸福区片改造步伐，实施胜利、慎礼等重点区片建设改造工程。加大城中村、城郊村改造力度，年内改造城中、城郊村62个，建设安置房260万平方米。实施轸大路、莱院路、通海东路等36项城区道路工程。组织开展“城市管理年”活动，搞好环境综合整治，提高城市“洁、绿、亮、美”水平。进一步理顺市区管理体制，完善长效化、规范化城市管理机制。坚持公交优先，整合公交资源，健全公共交通体系。强化城市水资源一体化管理，保障城市生产生活用水。加强城市管理信息化建设，扩大市区数字化城市管理覆盖范围。加大规划执法力度，逐步清理违章建筑，坚决制止乱搭乱建现象。

一体化推进镇村建设。加强社会主义新农村建设，推动公共财政向“三农”倾斜、公共设施向农村延伸、公共服务向农村覆盖、现代文明向农村传播，实现城乡统筹发展。年内基本实现全市城乡统筹一体发展规划、城镇总体规划和重要专项规划全覆盖。完成烟凤线福山段、上泽线牟平段改建等3项公路工程，启动国道309、龙口黄水线、成龙线牟平段改建和长岛南北长山大桥等5条公路项目，改造农村公路200公里，全市干线公路通车里程达到2200公里以上。加大农村住房建设和危房改造力度，完成农村住房建设面积380万平方米，改造危房8000户。加强重要水源地保护，搞好门楼水库、沐浴水库除险加固工程，全面完成12座中型水库和178座小型水库除险加固任务，对24个饮用水源地进行划界设标，维护水源安全。开展环境优美乡镇、生态文明村庄创建活动，促进农村环境持续改善。改造惠及200个村、15万人的农村自来水设施。加强环卫设施建设，推进城乡生活垃圾一体化处理。发挥龙口市在县域经济发展中的引领作用，支持各县市走特色、生态、高效的发展道路，推动县域经济扩规模、上水平。促进小城镇发展由数量规模型向质量效益型转变，全市新型农村社区覆盖率达到60%以上。

四、着力改善民生

坚持发展为了人民，发展依靠人民，发展的成果由人民共享，不断提高人民群众的幸福感、安全感和满意度，推动社会和谐稳定与长治久安。

(一)强化社会保障，切实解决好人民群众最关心、最直接、最现实的利益问题。

千方百计扩大就业。实施更加积极的就业政策，支持创业带动就业，鼓励自主创业、自谋职业。发挥好政府投资和重大项目带动就业的作用，引导和促进劳动密集型企业、中小企业、民营经济和服务业加快发展。落实国家、省有关减负稳岗政策，规范企业用工行为。加强基层劳动保障服务平台建设，加大公益性岗位开发力度，继续抓好城镇就业困难人员、高校毕业生、农民工、复退转业军人等重点群体就业，确保申报援助的零就业家庭“动态消零”。年内，创业培训6000人以上，开发公益性岗位4000个，帮扶困难群体再就业5000人以上，新增城镇就业12万人以上，农村劳动力转移就业2万人以上。

完善社会保障体系。强化社保扩面工作，将更多非公有制经济从业人员、灵活就业人员和农民工纳入社保覆盖范围。提高企业退休人员基本养老金。实行失业、工伤保险市级统筹，年内将2.1万名“老工伤”人员纳入工伤保险统筹范围。推进“金保工程”建设，免费为参保职工发放社会保障卡，年内发卡率达到70%以上。搞好国家首批新型农保试点工作，争取更多县市区列入试点范围。扎实做好五保供养工作，提高城乡低保标准。加快保障性安居工程建设，建设经济适用房5000套，对符合条件的低收入家庭实现廉租住房“应保尽保”。探索建立政府限价房等住房保障方式，有效解决较低收入群体的住房困难。

加大社会救助力度。继续开展“爱心捐助”活动，拓宽慈善救助资金来源渠道，做好困难家庭和困难学生救助工作。规范完善城乡医疗救助制度，进一步扩大救助范围。在全市建立优抚医疗“一站式”报销网络，实现新型优抚医疗保障制度全覆盖。加快县级社会福利中心建设，市老年福利服务中心、流浪未成年人救助保护中心年内投入使用。抓好社区居家养老工作，新建10处社区养老服务中心。

(二)繁荣社会事业，切实增强文化影响力和区域软实力。

发展文化事业和文化产业。加强社会主义核心价值体系建设，实施公民道德建设工程，深入开展群众性精神文明创建活动和“文明月”活动，提高市民文明素质和城市文明程度，巩固和发展全国文明城市创建成果。积极培育葡萄酒文化、红色文化、开埠文化、道教文化等特色文化品牌，搞好对外文化交流和城市形象宣传。加强文艺人才队伍建设，大力实施“精品工程”，推动文艺精品创作上水平。发挥好文化中心的引领作用，广泛开展“专业艺术演出季”、“社区文化乐万家”等文化活动，不断丰富群众文化生活。推进市博物馆新馆建设，抓好市县两级大型文化设施、乡镇综合文化站、农村文化大院、文化活动室和农家书屋建设。实施文化产业发展规划，促进传媒、出版印刷和创意等现代文化产业繁荣壮大。抓好动漫产业基地、五彩文化广场、辛安河科技文化公园等文化产业园区建设。

提高教育现代化水平。持续加大教育投入，全面提升基础教育和素质教育水平，大力发展高等教育、职业教育和民办教育。积极改善高校办学条件，支持驻烟高校搞好重点学科、重点实验室建设。加大中小学校布局调整力度，重视教师队伍建设，逐步解决农村小学部分学科教师不足问题，促进城乡教育均衡发展。改善中小学办学条件，加紧实施中小学校舍安全工程和农村中小学“两热一暖一改”工程，加快中小学图书馆(室)和网络教育“班班通”工程建设。健全学生资助政策体系，继续免除城乡义务教育书本费和杂费，解决好困难家庭子女上学问题。

提高公共卫生发展水平。推进医药卫生体制改革，扩大基本医疗保障覆盖面，落实国家基本药物制度，促进基本卫生服务均等化。将城镇居民医保补助标准、新农合补助标准均提高到每人每年100元。加强公共卫生服务能力建设，逐步改善专业公共卫生机构的设施条件，提高疾控机构实验室装备水平。规范城市社区卫生服务，做好政府购买社区公共卫生服务工作。加强基层卫生服务网络建设，完成20个乡镇卫生院、500所村卫生室标准化改造。加大甲型H1N1流感、手足口病等重点传染病防控力度，增强应对突发公共卫生事件的能力。加快中医药事业发展，加强中医特色专科建设，推广中医药适宜技术。

积极发展其他社会事业。认真贯彻落实《全民健身条例》，搞好城乡公共体育设施建设，广泛开展全民健身活动。提高竞技体育水平，积极参加第二十二届省运会，筹备好第三届亚沙会。加强人口计生基层基础工作，稳定低生育水平。继续支援四川地震灾区恢复重建，积极帮扶栖霞，支持长岛科学发展，搞好援藏、援疆工作，抓好定陶烟台工业园建设。关心支持老龄、妇女、儿童、残疾人和红十字事业，做好外事侨务、民族宗教、史志档案、防震减灾、国防教育、人民防空、民兵预备役、双拥共建等工作。

(三)加强社会管理，切实维护和谐稳定的良好局面。

加强民主法制建设，积极开展法治城市、法治县市区创建活动，认真做好法律服务、法律援助和法律宣传工作，搞好城乡基层民主政治建设，保障人民群众更好地行使对政府工作的知情权、参与权和监督权。深入开展平安创建活动，完善社会治安防控体系，依法防范和打击违法犯罪活动，努力构建和谐稳定模范城市。建立健全社会稳定风险评估和大调解工作机制，积极预防化解社会矛盾。做好失地农民就业安置和城镇拆迁、旧城改造等工作，进一步形成依法有序表达诉求、及时有效解决问题的社会环境。开展“安全生产基层基础建设年”活动，完善安全生产长效机制，从根本上减少和遏制各类事故发生。健全粮食调控体系，抓好地方储备粮油管理，保障市场供应。加强物价监测预警，保持市场价格稳定。高度重视食品药品安全，提升食品药品安全水平。健全完善应急管理体系，建立海洋气象监测预警基地，防范抵御极端气候灾害。推进社会管理创新，解决好流动人口服务管理、特殊人群帮教管理、社会组织管理服务等问题，不断提高社会管理的科学化水平，让群众生活更安全、更方便、更文明、更和谐。

五、加强政府自身建设

保持平稳较快发展，夺取应对国际金融危机冲击的全面胜利，对政府工作提出了新的更高要求。我们必须用时代发展的眼光审视政府工作，用改革创新的精神加强自身建设，解放思想，奋发进取，努力建设为民、务实、清廉政府，推动政府工作全面上水平。

(一)积极创新工作思路，加快转变政府职能。深入学习实践科学发展观，不断增强科学判断形势、把握发展机遇、破解发展难题和应对复杂局面的能力，积极探索新形势下改进政府工作、推动科学发展的新思路、新办法。加强对经济社会发展的战略性研究，高质量编制国民经济和社会发展“十二五”规划。完成市、县两级政府机构改革任务，科学梳理整合部门职能，推进政企分开、政资分开、政事分开、政府与市场中介组织分开。进一步理顺事权，推动市级管理权限下放，强化政府服务职能。建立健全决策咨询机制，完善重大决策的社会参与、专家咨询、听证公示和决策后评估、责任追究制度。综合运用规划、财政、信贷、土地、环保等手段加强经济调控管理，推动结构调整和产业升级，提高科学发展水平。

(二)扎实推进依法行政，不断优化发展环境。继续深化行政审批制度改革，实施网上行政审批和行政审批电子监察，扩大并联审批、告知承诺、联合年检等改革措施的实施范围。发展电子政务，推动政务信息资源开发和共享，提升网上服务和跨部门协同服务水平。强化行业作风建设，加强纠风专项治理，坚决纠正损害群众利益的不正之风。深化行政执法体制改革，规范执法程序，提高执法水平。创新政务服务手段，建立健全政府联系企业、服务企业的长效机制。加强行政复议工作，改进行政复议方式方法。积极推行政府信息公开，打造阳光政府。认真接受市人大及其常委会的法律监督和工作监督，坚持定期向市人大常委会报告工作。认真接受市政协的民主监督，加强与市政协的工作沟通和协商。主动听取各民主党派、人民团体和社会各界的意见和建议，自觉接受社会公众监督和舆论监督。办理好人大代表建议和政协提案，提高工作质量和实效。

(三)牢固树立宗旨意识，始终坚持执政为民。坚持以人为本，正确行使人民赋予的权力，保护好群众的正当利益。树立牢固的公仆意识和正确的政绩观，坚持问政于民、问需于民、问计于民，多办顺民意、解民忧、增民利的实事，多做谋长远、打基础、利全局的工作，杜绝劳民伤财的面子工程、形象工程。转变工作作风，深入基层和群众，进一步密切党和政府同人民群众的血肉联系。继续组织实施为民服务实事工程，让广大群众更多地分享改革发展的成果。

(四)坚决抓好廉政建设，努力打造良好形象。坚持标本兼治、综合治理、惩防并举、注重预防的方针，严格落实党风廉政建设责任制，深入开展廉政风险防范管理工作，扎实推进惩治和预防腐败体系建设。深化行政监察和审计监督，加大查办案件和行政问责力度，着力解决重点领域的腐败问题，严肃查处滥用职权、贪污贿赂、腐化堕落、失职渎职等各类案件。发扬艰苦奋斗精神，树立过紧日子的思想，坚决反对讲排场、比阔气、铺张浪费和奢侈之风，严格控制各类行政经费，把有限的资金真正用在促进发展、改善民生急需的地方。加强教育、引导和管理，努力建设政治坚定、业务精通、清正廉洁、作风正派的公务员队伍。

各位代表！新的一年，应对危机挑战、推动科学发展的任务光荣而艰巨。让我们更加紧密地团结在以胡锦涛同志为总书记的党中央周围，高举中国特色社会主义伟大旗帜，以邓小平理论和“三个代表”重要思想为指导，深入贯彻落实科学发展观，在中共烟台市委的坚强领导下，解放思想、迎难而上，锐意进取、扎实工作，为全面完成“十一五”规划确定的各项任务目标、建设文明富庶新烟台而努力奋斗！
